# Left Ventricular Deformation and Myocardial Work Parameters in Patients with Hereditary Transthyretin Amyloidosis Treated with Patisiran: A Single-Center Study

**DOI:** 10.3390/jcm13164914

**Published:** 2024-08-20

**Authors:** Daniela Di Lisi, Francesco Comparato, Antonella Ortello, Vincenzo Di Stefano, Filippo Brighina, Francesca Macaione, Giovanni La Fiura, Leandro Di Caccamo, Cristina Madaudo, Alfredo R. Galassi, Giuseppina Novo

**Affiliations:** 1 Division of Cardiology, University Hospital Paolo Giaccone, 90127 Palermo, Italy; 2 Department of Health Promotion, Mother and Child Care, Internal Medicine and Medical Specialties (PROMISE) “G. D’Alessandro”, University of Palermo, 90127 Palermo, Italy; 3 Section of Neurology, Department of Biomedicine, Neuroscience and Advanced Diagnostic (BIND), University of Palermo, 90127 Palermo, Italyfilippo.brighina@unipa.it (F.B.)

**Keywords:** cardiac amyloidosis, patisiran, speckle tracking echocardiography, transthyretin

## Abstract

**Background:** In recent years, many advances have been made in the treatment of hereditary transthyretin amyloidosis (ATTRv). Patisiran is a small-interfering RNA used to treat ATTRv with only polyneuropathy or polyneuropathy and cardiomyopathy. The aim of our study was to assess the effect of patisiran on cardiac function in ATTRv patients using speckle tracking echocardiography (STE) analysis. **Methods**: A single-center prospective study was performed enrolling 21 patients with ATTRv (11 M—52% of the population; 10 F—48% of the population; median age 66 ± 8.4 years old). A total of 7 patients had cardiac amyloidosis and polyneuropathy, and 14 patients had only polyneuropathy without cardiac involvement. Cardiological evaluation including electrocardiograms, echocardiography with STE, and assessment of myocardial work parameters was performed in all patients before starting patisiran and after 9–18 months. Functional capacity was assessed using the 6 min walk test; quality of life was assessed using the Kansas City Cardiomyopathy Questionnaire (KCCQ). **Results**: We did not find a significant difference in gender prevalence of ATTR amyloidosis in all of the population (*p*-value 0.79), but we found that cardiac amyloidosis significantly predominated in the male sex compared to patients with only neuropathy. In all patients, we found a slight improvement in functional capacity and quality of life. We did not find significant changes in left ventricular ejection fraction (LVEF), but we found a significant improvement in left ventricular global longitudinal strain (GLS), global work waste (GWW), and global work efficiency (GWE), especially in patients with cardiac amyloidosis; E/e’ average and left atrial stiffness also improved significantly in patients with cardiac amyloidosis. **Conclusions:** Our study confirms a positive effect of patisiran on cardiac function, particularly the absence of signs of subclinical deterioration as detected by very sensitive STE parameters such as GLS, MW, and atrial stiffness during follow up in patients treated with patisiran.

## 1. Introduction

Hereditary transthyretin amyloidosis (ATTRv) is a rare, inherited disorder driven by pathogenic variants in the transthyretin (TTR) gene, leading to organ involvement [1,2]. Cardiac amyloidosis can result in heart failure with preserved ejection fraction (HFpEF), necessitating consideration during diagnosis [3,4]. The condition exhibits notable genotype–phenotype variability, with mutations manifesting in polyneuropathy, cardiomyopathy, or a mixed phenotype [5,6,7]. Some carriers may remain asymptomatic or develop symptoms later, emphasizing the need for ongoing monitoring [8,9].

Early diagnosis is crucial due to advancements in treatment, targeting TTR production, stabilization, and amyloid removal [10,11]. Therapeutic interventions, including TTR stabilizers like tafamidis and RNA interference drugs such as patisiran or inotersen, offer significant promise [12]. Patisiran’s efficacy was demonstrated in the APOLLO trial, where it reduced cardiac symptoms and improved quality of life and ambulatory capacity compared to placebo [13]. Subsequent analysis showed a preservation of cardiac function and reduced adverse events, indicating its potential to halt or reverse cardiac manifestations of ATTRv [14,15].

The favorable effects of patisiran compared to placebo were observed in all subgroups defined by demographic data and baseline characteristics of ATTRv. In the prespecified cardiac subpopulation, significant positive effects of patisiran were observed for certain exploratory endpoints related to cardiac biomarkers and echocardiographic measures (left ventricular wall thickness, left ventricular longitudinal strain).

The aim of our study was to confirm patisiran’s protective effect on cardiac function in ATTRv patients, and to explore its impact on advanced echocardiographic parameters of cardiac deformation such as left ventricular global longitudinal strain (GLS), myocardial work indices, and atrial strain.

## 2. Materials and Methods

This is a prospective single-center study performed enrolling 21 patients with ATTRv followed at the Cardiomyopathy Outpatient Clinic of the Cardiology Division at the “Paolo Giaccone” University Hospital in Palermo, in collaboration with the Neurology Clinic of the same Hospital, during the period 2020–2023. Data were analyzed 9 (T1) and 18 months (T2) after starting therapy with patisiran.

Inclusion criteria were the following:

- Age ≥ 18 years old;
- Hereditary transthyretin amyloidosis with polyneuropathy with or without cardiomyopathy;
- Signed informed consent for participation in this study.

Exclusion criteria were the following:

- Other forms of non-amyloidotic heart disease;
- Non-mutational transthyretin-related cardiac amyloidosis;
- Other forms of non-amyloidotic neuropathy;
- Patients carrying a mutation in the gene encoding transthyretin without clinical manifestations (cardiac and/or neurological);
- Multiple myeloma and/or other cancer.

All patients enrolled had a mutation in the gene encoding for transthyretin (TTR). These patients were divided into two phenotypic groups based on the clinical manifestations of the disease:

- Hereditary transthyretin amyloid cardiomyopathy with neuropathy: Group A;
- Hereditary transthyretin amyloidosis with only neuropathy: Group B.

Before starting patisiran, patients underwent a clinical and instrumental evaluation that included the following:

- Cardiology examination with comprehensive medical history collection assessing the presence of cardiovascular risk factors (arterial hypertension, dyslipidemia, diabetes mellitus, cigarette smoking, obesity) and cardiovascular comorbidities (including ischemic heart disease, peripheral artery diseases, transient ischemic attacks, and strokes); measurement of blood pressure and recording of main anthropometric/functional variables (weight, height, BMI, BSA, NYHA);
- ECG (electrocardiogram) with assessment of rhythm, atrial abnormalities, atrioventricular and intraventricular conduction anomalies (atrioventricular blocks, bundle branch blocks, and hemiblocks), pseudonecrosis waves, arrhythmias (e.g., atrial fibrillation);
- Laboratory test with complete blood count, urea, creatinine, potassium, sodium, c-TnT-hs (high-sensitivity cardiac troponin T), AST (aspartate aminotransferase), ALT (alanine aminotransferase), total cholesterol, HDL (high-density lipoprotein), LDL (calculated by the Friedewald formula), triglycerides, blood glucose, and NT-proBNP (N-terminal pro-B-type natriuretic peptide);
- Color-Doppler echocardiography with assessment of conventional echocardiographic parameters and myocardial deformation indices of the left ventricle and left atrium; assessment of myocardial work parameters;
- Bone scintigraphy with diphosphonates assessing cardiac uptake according to the Perugini scale;
- Cardiac magnetic resonance imaging with and without contrast agent, evaluating T1-T2 mapping and extracellular volume (ECV);
- Six-minute walk test to assess functional capacity;
- Assessment of quality of life was conducted through the completion of the Kansas City Cardiomyopathy Questionnaire (KCCQ);
- Neurological evaluation, including a comprehensive general neurological examination (tests to assess muscle strength, reflexes, sensitivity, and coordination), electromyography, and electroneurography to evaluate peripheral nervous system function and integrity, along with assessment of cognitive and sensory functions (memory, attention, language, and other cognitive functions, as well as sensory sensitivity).

The diagnosis of cardiac ATTR amyloidosis was performed according to the position statement of the European Society of Cardiology [9]. It was established by meeting echocardiographic and/or cardiac magnetic resonance imaging criteria, combined with a positive uptake of the radiotracer in scintigraphy using 99mTc-pyrophosphate (PYP), 99mTc -3,3-diphosphono-1,2-propanodicarboxylic acid (DPD), or 99mTc-hydroxymethylene diphosphonate (HMDP) with Grade ≥ 2 according to the Perugini score, in the absence of a monoclonal component in the blood sample.

The diagnosis of amyloidotic neuropathy was made by neurologists and based on the presence of pathognomonic neurological signs and symptoms.

Cardiological and neurological consultations were performed 9 (T1) and 18 months (T2) after starting patisiran. Cardiological evaluation during follow-up included the following: color-Doppler echocardiography with speckle tracking analysis, and assessment of functional capacity and quality of life.

### 2.1. Echocardiography Evaluation

Echocardiographic examination was performed according to the American Society of Echocardiography/European Association of Cardiovascular Imaging recommendations for chamber quantification and diastolic function evaluation [16,17].

The left ventricular ejection fraction (LVEF) was calculated using the Simpson biplane method from the apical four-chamber and two-chamber views. The left atrial volume was determined using the area-length method in both apical four-chamber and two-chamber views and was indexed to body surface area. In addition, the three-dimensional ejection fraction was also measured. The systolic function of the right ventricle was assessed by measuring the tricuspid annular plane systolic excursion (TAPSE) and the S’ wave was measured with tissue Doppler imaging (TDI) at the free wall of the right ventricle near the tricuspid annulus. Criteria for assessing left ventricular diastolic dysfunction were in accordance with the recommendations of the EACVI/ASE [16].

The assessment of 2D strain was conducted using commercially available software from GE (Echopac V.202, GE) employing a speckle tracking echocardiography (STE) methodology. The analysis was performed offline on images acquired in all three apical views (four-chamber, two-chamber, and three-chamber), with a frame rate ranging between 60 and 80 frames per second (fps). The assessment of left ventricular strain involves the operator manually tracing the endocardial border, marking points (speckles) with specific acoustic features, which are then consolidated by the software. The endocardial border of the left ventricle was traced during the end-systole and the region of interest (ROI) was refined to exclude the pericardium, meticulously aligning the epicardial border. We measured the left ventricular global longitudinal strain (GLS), derived from the strain calculated in the three apical views. A GLS value of −21.5 ± 2% was deemed normal, with a lower limit of the normal range (LLN) of −18% [17].

In addition, we assessed the apex-to-base ratio of longitudinal strain (SAB: average apical longitudinal strain/average basal longitudinal strain) and relative apical sparing (RAS: average apical longitudinal strain/sum of the average basal and mid longitudinal strain) [18,19,20,21].

The analysis of left atrial strain using STE was conducted following the same principle used for the other cardiac chambers and utilizing the same software. Two-dimensional echocardiographic images were acquired in the apical four-chamber and two-chamber views, with a frame rate ranging from 40 to 80 frames per second, considering three consecutive cardiac cycles. In this case, manual tracking of the endocardial border of the left atrial wall was used. The epicardial tracking was automatically generated by the system through the creation of a region of interest (ROI). In this study, the normal value of reservoir strain (PALS) was considered as 42.5% (IQR 36.1–48.0%), with a lower limit of normal (LLN) of 26.1 ± 0.7%. Additionally, atrial stiffness was calculated from the ratio between the E/e’ average and PALS [22].

The calculation of myocardial work was performed using specific software provided by GE (Echopac V.202, GE). To calculate the global work index (GWI), the fundamental physical principle was applied wherein the area under the PSL curve, recorded via STE, was multiplied by the brachial arterial pressure value (representing the force exerted by the cardiac muscle). Hence, the measured values of arterial pressure were inputted into the software at the beginning of the acquisitions. Calculations were obtained from a representative cardiac cycle, specifically identifying the timing of mitral and aortic valve closure and opening. In addition to GWI, we assessed global constructive work (GCW), global work waste (GWW), and global work efficiency (GWE).

### 2.2. Statistical Analysis

Data were recorded in an Excel spreadsheet format (.xls), which constituted the database used for this scientific study. Statistical analyses were performed using R Software (R version 4.3.3).

For data presentation, categorical variables are reported as the absolute frequency (n°) and relative percentage to the total (%), while continuous variables are described as the mean ± standard deviation (SD). The analysis of statistical significance was conducted using the paired Student’s *t*-test for variables following a normal distribution and the Wilcoxon signed-rank test for paired variables that do not follow a normal distribution.

To assess differences among multiple groups, since not all variables included in the analysis adhered to the assumption of normality, two types of tests were used: for normally distributed data, an ANOVA model was employed, while the non-parametric Kruskal–Wallis test was used for non-normally distributed data. These tests were used to compare more than two independent samples in terms of population.

To evaluate the statistical significance of qualitative variables, the χ^2^ test for proportions was used. A significance level of alpha less than 0.05 was considered statistically significant.

## 3. Results

The sample population consisted of 21 patients (11 M, 10 F; median age 66 ± 8.4 years old). 

The general characteristics of the study population are summarized in Table 1. Seven patients (33.4%) had cardiac amyloidosis and amyloid neuropathy and fourteen patients (66.6%) had amyloid neuropathy alone. In the analyzed sample, the mutations were distributed as follows: (a) the “Pheu64Leu” mutation was most frequent, present in 15 patients (71.4%); (b) Val122Ile was present in 2 patients (9.5%); (c) Glu89Gln was present in 2 patients (9.5%); (d) His110Asn was present in only 1 patient (4.7%); and (e) Ser97Phe was also present in only 1 patient (4.7%). Among the patients with only neurological involvement, 11 had the Phe64Leu mutation, 2 had the Val122Ile mutation, and 1 had the His110Asn mutation. Within the population affected by both cardiac and neurological amyloidosis, all subjects were male, and among neuropathic subjects, 4 were male and 10 were female.

We did not find a significant difference in gender prevalence of ATTR amyloidosis in all of the population (*p*-value = 0.79), but we found that cardiac amyloidosis significantly predominated in the male sex compared to patients with only neuropathy (Table 2).

Among patients with amyloidotic neuropathy and/or cardiac amyloidosis, 21 (67.7%) were undergoing treatment with patisiran; only 3 patients with cardiac amyloidosis had been treated with tafamidis for approximately 1 year before starting patisiran therapy.

The characteristics of the study population divided by phenotypic classes are summarized in Table 2.

A proportion of 42.9% of patients had well-controlled arterial hypertension, 47% of patients had dyslipidemia, and 4.8% had diabetes mellitus (Table 1).

Patients with the Phe84Leu genetic mutation had a negative bone scintigraphy according to the study by Musumeci et al., which demonstrated a low sensitivity of this test in identifying cardiac amyloidosis caused by such a mutation [23]. The mechanism underlying this phenomenon is unclear, but it is hypothesized to be related to a lower presence of calcium in amyloid fibrils associated with this mutation.

Analyzing echocardiographic data, we confirmed in patients with cardiac amyloidosis the typical alterations: concentric left ventricular hypertrophy with higher values of interventricular septum (IVS), posterior wall (PW), and relative wall thickness (RWT) compared to patients with only neuropathy.

### 3.1. Results Pre- and Post Treatment with Patisiran in Neuropathic Patients

During the follow-up of patients affected by amyloidotic neuropathy undergoing patisiran therapy, clinical and echocardiographic parameters were compared. The results are reported in Table 3.

Concerning clinical parameters, we found a mild improvement in functional capacity in the 6 min walking test and an improvement in quality of life, evaluated by the KCCQ score, with a mean improvement of at least 10 points.

From the analysis of the results at different follow-up time points, no patient developed cardiac amyloidosis and the echocardiographic parameters remained stable during the therapy. In fact, we did not find significant changes in E/e’ average values, GLS (−20.4 ± 3.57 at T0 vs. −19.69 ± 3.81 at T1, *p*-value = 0.61; vs. −19.88 ± 2.69 at T2, *p*-value = 0.66), GWI, GWW, GWE, or left atrial stiffness (T0: 0.45 ± 0.43 vs. T1: 0.56 ± 0.51, *p*-value = 0.54; vs. T2: 0.54 ± 0.5, *p*-value = 0.61).

We also observed a slight increase in the apical-to-basal strain ratio SAB (1.58 ± 0.27 at T0 vs. 1.68 ± 0.62 at T1, *p*-value = 0.58; vs. 1.76 ± 0.22 at T2, *p*-value = 0.06) and relative apical sparing (the latter being statistically significant at T2; 0.71 ± 0.09 at T0 vs. 0.88 ± 0.2 at T2, *p*-value = 0.0075), although these values remained within the normal range.

### 3.2. Results Pre- and Post Treatment with Patisiran in Patients with Cardiomyopathy

During the follow-up of patients affected by cardiac amyloidosis as well as neuropathy undergoing patisiran therapy, clinical and echocardiographic parameters were compared between T0, T1, and T2. The results are reported in Table 4.

**Figure 1:**
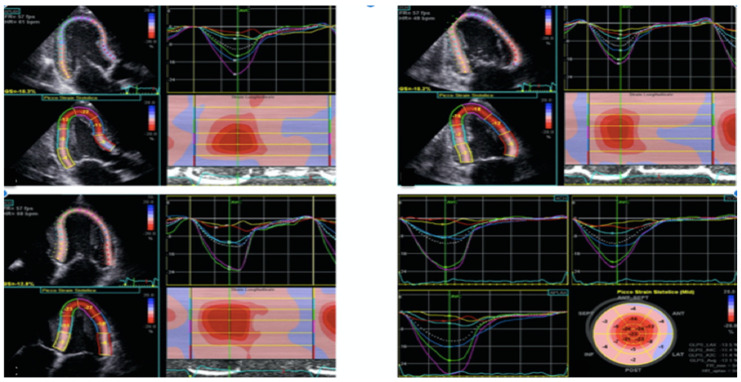
Global longitudinal Strain in patients with cardiac amyloidosis, baseline assessment.

**Figure 2:**
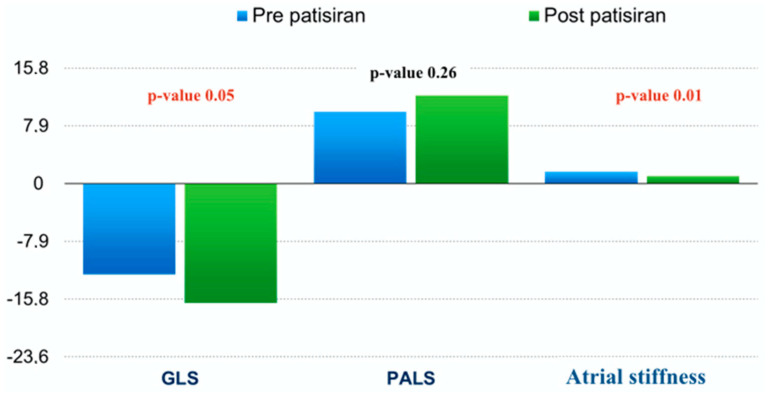
GLS, PALS, and atrial stiffness variations in patients undergoing therapy with patisiran and cardiac amyloidosis. GLS: global longitudinal strain. PALS: peak atrial longitudinal strain.

**Figure 3:**
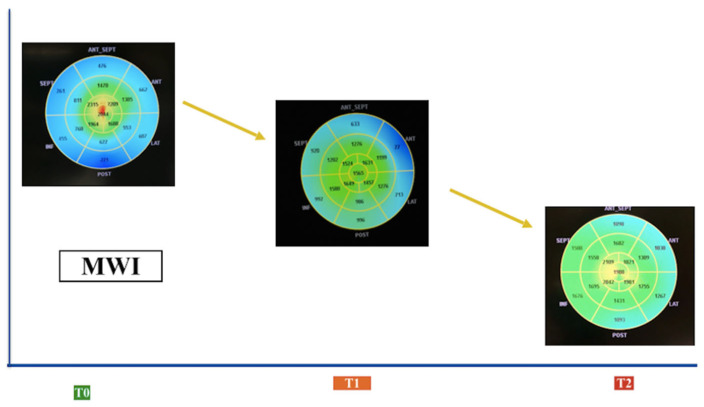
Variations in MW indices (MWIs) in patients with cardiac amyloidosis treated with patisiran.

Patients experienced a mild improvement in functional capacity assessed through the walking test and KCCQ. We found a non-significant reduction in NT-proBNP values (respectively, at T0 and T2: 1956 ± 3297 pg/mL and 764 ± 453 pg/mL, *p*-value = 0.36). Echocardiographic analysis showed a significant improvement in E/e’ average values at T1 and T2 (T0: 15 ± 5 vs. T1: 10.3 ± 3, *p*-value = 0.05; vs. T2: 10.2 ± 2.5, *p*-value = 0.04), GLS at T2 (T0: −13 ± 5 vs. T2: −17.2 ± 1.29, *p*-value = 0.05), GWW at T1 and T2 (T0: 162 ± 95 mmHg% vs. T1: 76 ± 35 mmHg%, *p*-value = 0.04; vs. T2: 64 ± 8 mmHg%, *p*-value = 0.01) and GWE at T2 (T0: 88 ± 6% vs. T2: 94 ± 2%, *p*-value = 0.02), as well as left atrial stiffness at T1 and T2 (T0: 2 ± 1 vs. T1: 1 ± 0.66, *p*-value = 0.04; vs. T2: 1 ± 0.6, *p*-value = 0.04); see Figure 1, Figure 2 and Figure 3. The apical-to-basal longitudinal strain ratio decreased, albeit not statistically significantly (respectively, at T0 and T2: 3.3 ± 2.1 and 2.17 ± 1, *p*-value = 0.22), suggesting a potential greater improvement in longitudinal strain values at the basal segments of the left ventricle.

The other echocardiographic parameters did not show significant changes during the follow-up. Only TAPSE decreased significantly. Rather, there was stability observed in the wall thickness and LVEF, suggesting a potential slowdown in disease progression.

In patients treated with patisiran who underwent reevaluation through cardiac magnetic resonance imaging (only 20% of patients), we did not observe progression of cardiac disease (no increase in left ventricular mass, no increase in delayed enhancement during follow-up). Unfortunately, the data are inclusive considering the small number of patients.

## 4. Discussion

Patisiran is a small interfering RNA (siRNA) drug which revolutionized the treatment of hereditary ATTR amyloidosis. Currently, patisiran is indicated for the treatment of patients with ATTRv cardiac amyloidosis and concurrent polyneuropathy or only ATTRv polyneuropathy alone (stage 1 or 2) [24,25].

Two trials showed a positive effect of patisiran on cardiac function and an improvement in quality of life and functional capacity [14,15,16].

Our results are in accordance with the literature data and results of Apollo trials [26]. In fact, we found a positive effect of patisiran in both ATTRv patients with cardiac amyloidosis plus polyneuropathy and ATTRv patients with only polyneuropathy.

None of the patients treated with patisiran for polyneuropathy alone, despite exhibiting mutations primarily associated with mixed and/or cardiac manifestations (Val142Ile and Phe64Leu), developed cardiac amyloidosis during the follow-up period; moreover, all echocardiographic parameters remained stable in these patients, indicating no progression of the disease. Thus, patisiran could also have a stabilizing role at the cardiac level, not only at the neurological level, and in patients with only neuropathy, other factors, such as the type of ATTR mutation, could influence the development of cardiomyopathy.

Probably, patisiran could slow the development of cardiac disease and the progression of amyloid deposits in ATTRv patients with only neuropathy, but much more extensive data are needed to confirm this hypothesis. Functional capacity assessed using the 6 min walking test and quality of life assessed by KCCQ improved in all patients during treatment, even in patients with neuropathy alone.

In patients with cardiac amyloidosis and neuropathy, we found an improvement in hemodynamic compensation, as demonstrated by a statistically significant reduction in E/e’ average ratio and in NT-proBNP values.

In addition, in patients with cardiac amyloidosis, treatment with patisiran was associated with a trend for improvement in GLS after 18 months. These results are consistent with those obtained in the APOLLO trial. In addition, however, our study assessed changes in myocardial work and showed a significant reduction in GWW after 9–18 months and a significant improvement in GWE after 18 months, demonstrating how the drug enhances the efficiency of the left ventricle’s work. We did not find significant changes in LVEF during follow-up or in other conventional echocardiographic parameters.

It is known that global longitudinal strain is more sensible than LVEF in detecting early subclinical cardiac dysfunction, but it is influenced by loading conditions [27]. Myocardial work indices are not influenced by loading conditions [28], and they have multiple clinical applications such as in patients with heart failure [29]. In patients with cardiac amyloidosis, MW could help in identifying early cardiac damage and/or monitoring the response to therapy [30]. In addition, GCW may be of additional prognostic value to LVEF and GLS in predicting heart failure hospitalization and all-cause mortality in patients with cardiac amyloidosis [31].

It is well known that atrial involvement is very precocious in patients with cardiac amyloidosis, leading to electromechanical dissociation of the atrium and increasing the risk of thromboembolism even in patients without atrial fibrillation. The assessment of atrial stiffness and PALS in patients with cardiac amyloidosis could be useful in the early detection of atrial involvement and in guiding the indication to anticoagulation therapy even in patients in sinus rhythm. In our series of patients, atrial stiffness improved already after 9 months of treatment with patisiran.

According to our results, a multiparametric echocardiographic evaluation using STE parameters, giving a more comprehensive evaluation of cardiac involvement, is useful in patients with ATTRv in making an early diagnosis of cardiac involvement, in promptly starting treatment, and in monitoring the response to therapy. Thus, echocardiography is a simple tool to use in the diagnosis and monitoring of patients with cardiac amyloidosis, and it should be performed in echocardiographic laboratories with expertise [32].

## 5. Conclusions

Our study confirms the positive effect of patisiran on cardiac function in ATTRv either in patients with only polyneuropathy or in patients with polyneuropathy and associated cardiac amyloidosis.

Particularly, patisiran seems to have a stabilizing role in patients with only polyneuropathy; in patients with cardiomyopathy, patisiran was associated with a significant improvement in myocardial deformation indices (GLSs), atrial stiffness, and myocardial work indices.

Thus, we argue that measuring STE parameters (GLS, MW, and atrial stiffness) for a more comprehensive evaluation of cardiac function could be of aid in ATTRv for the early diagnosis of cardiac involvement and monitoring. Larger studies are necessary to confirm our data.

## 6. Study Limitations

The main limitation of our study is the small sample size; however, this limitation is understandable given the rarity of the disease and monocentric nature of this study. We aim to confirm our data in a larger multicentric study. Another limitation is the absence of T1 and T2 mapping CMR data due to the heterogeneous provenance of patients who sometimes underwent MRI in centers where this analysis was not available.

## Figures and Tables

**Table 1:** General and clinical characteristics of the study population. BMI: body mass index; ACE-I: angiotensin-converting enzyme inhibitor; ARB: angiotensin receptor antagonist.

Features		Value
Sex n° pts (%)	Male	11 (52%)
	Female	10 (48%)
Age (mean y.o. ± DS)	66 ± 8	
BMI (mean Kg/mq ± DS)		26 ± 7
	Arterial hypertension	9 (42.9%)
Cardiovascular risk factors n° patients (%)	Diabetes mellitus	1 (4.8%)
	Dyslipidemia	10 (47%)
	Smoking	3 (14%)
	Family history of CVD	6 (28%)
	Other CV comorbidities	5 (24%)
Therapy n° patients (%)	Beta-blocker	9 (42.9%)
	Calcium channel blocker	4 (19%)
	ACE-i/ARB	5 (23%)
	Antiplatelet agent	7 (33%)
	Diuretic	3 (14%)
	Statins	7 (33%)
	Ezetimibe	1 (4.8%)
	Patisiran	17 (80.95%)
	Tafamidis	3 (14.29%)

**Table 2:** Characteristics of patients divided by phenotypic classes. BMI: body mass index.

Features	Cardiac Amyloidosis (7 Pts)	Neuropathy (14 pts)	*p*-Value
Male n° patients (%)	7 (100%)	4 (29%)	0.0027
Female n° patients (%)	0 (0%)	10 (71%)	0.0027
Age (value ± DS)	66 ± 8.4	65.7 ± 7.8	0.93
BMI (Kg/mq)	26 ± 5.6	26 ± 7.4	1
Hypertension n° patients (%)	3 (42.86%)	6 (46%)	0.89
Diabetes mellitus n° patients (%)	0 (0%)	1 (7.69%)	0.46
Dyslipidemia n° patients (%)	2 (28.5%)	7 (53.8%)	0.28
Smoking n° patients (%)	1 (14.29%)	2 (15.38%)	0.94
Family history of CVD n° patients (%)	1 (14.29%)	5 (38.5%)	0.26
Other cardiovascular comorbidities n° patients (%)	1 (14.29%)	4 (30.7%)	0.42

**Table 3:** Clinical and echocardiographic parameters pre- and post treatment with patisiran in neuropathic patients. GCW: global constructive work; GLS: global longitudinal strain; GWI: global work index; GWE: global work efficiency; GWW: global work waste; IVS: interventricular septum; LA: left atrium; LVEF: left ventricular ejection fraction; NT-pro BNP: N-terminal prohormone of brain natriuretic peptide; PW: posterior wall; SAB ratio: apical–basal strain ratio; TAPSE: tricuspid annular plane excursion; Tnt: troponin.

Echocardiographic Parameters	Neuropathy T0	Neuropathy T1 (*p*-Value)	Neuropathy T2 (*p*-Value)
IVS (mm)	10.21 ± 2.12	10.69 ± 1.84 (0.52)	11 ± 2 (0.31)
PW (mm)	8.86 ± 1.83	9.54 ± 1.61 (0.30)	10 ± 1 (0.051)
LVEF 2D (%)	60.14 ± 5.25	59.23 ± 4.95 (0.64)	57 ± 4 (0.08)
End diastolic volume (mL/mq)	77.5 ± 20.3	80.23 ± 23.34 (0.74)	89 ± 29 (0.23)
S’ septal (cm/s)	9.33 ± 2.99	8 ± 1.8 (0.16)	8 ± 3 (0.25)
S’ lateral (cm/s)	10.67 ± 3.08	8.62 ± 2.60 (0.06)	9 ± 4 (0.22)
E/e’ mean	8.22 ± 3.89	9.52 ± 4 (0.35)	11 ± 7 (0.19)
TAPSE (mm)	22.31 ± 4	20.62 ± 1.8 (0.16)	21 ± 3 (0.33)
LA vol Biplane (mL)	58.93 ± 25.10	62.69 ± 21.76 (0.67)	64 ± 14 (0.51)
GLS LV 2D (%)	−20.40 ± 3.57	−19.69 ± 3.81 (0.61)	−19.88 ± 2.69 (0.66)
PALS (%)	24.65 ± 11.35	25.21 ± 1.36 (0.85)	20 ± 5 (0.17)
Atrial Stiffness	0.45 ± 0.43	0.56 ± 0.51 (0.54)	0.54 ± 0.5 (0.61)
GWI (mmHg%)	1911 ± 408	1830 ± 402 (0.60)	1800 ± 145 (0.34)
GCW (mmHg%)	2293 ± 474	2168 ± 462 (0.48)	2100 ± 162 (0.16)
GWW (mmHg%)	78.33 ± 47.51	109.18 ± 79.85 (0.22)	68 ± 40 (0.53)
GWE (%)	95.78 ± 2.05	94.45 ± 2.46 (0.12)	95 ± 2 (0.31)
SAB ratio	1.58 ± 0.27	1.68 ± 0.62 (0.58)	1.76 ± 0.22 (0.06)
Relative apical sparing	0.71 ± 0.09	0.70 ± 0.17 (0.84)	0.88 ± 0.2 (0.0075)
NT-proBNP (pg/mL)	264 ± 401	183 ± 200 (0.5)	269 ± 300 (0.97)
Hs TnT (ng/L)	4.47 ± 5.4	1.6 ± 3 (0.09)	1.5 ± 1.7 (0.06)

**Table 4:** Clinical and echocardiographic parameters pre- and post treatment with patisiran in patients with cardiomyopathy. GCW: global constructive work; GLS: global longitudinal strain; GWI: global work index; GWE: global work efficiency; GWW: global work waste; IVS: interventricular septum; LA: left atrium; LVEF: left ventricular ejection fraction; NT-pro BNP: N-terminal prohormone of brain natriuretic peptide; PW: posterior wall; SAB ratio: apical–basal strain ratio; TAPSE: tricuspid annular plane excursion; Tnt: troponin.

Echocardiographic Parameters	Cardiac Amyloidosis T0	Cardiac Amyloidosis T1 (*p*-Value)	Cardiac Amyloidosis T2 (*p*-Value)
IVS (mm)	17 ± 4	17.25 ± 3.30 (0.85)	17 ± 3 (1)
PW (mm)	15 ± 3	14.5 ± 2.08 (0.72)	15 ± 2 (1)
LVEF 2D (%)	52 ± 7	52 ± 3.56 (1)	52 ± 4 (1)
End diastolic volume (mL/mq)	93 ± 29	106 ± 18 (0.33)	104 ± 24 (0.45)
S’ septal (cm/s)	81 ± 25	97.2 ± 20 (0.21)	94 ± 21 (0.31)
S’ lateral (cm/s)	5 ± 2	4.75 ± 0.96 (0.77)	6 ± 1 (0.25)
E/e’ mean	7 ± 2	6.5 ± 1 (0.56)	7 ± 1 (1)
TAPSE (mm)	15 ± 5	10.3 ± 3 (0.05)	10.2 ± 2.5 (0.04)
LA vol Biplane (mL)	18 ± 6	20.5 ± 4.6 (0.39)	20 ± 4 (0.47)
GLS LV 2D (%)	−13 ± 5	−14.6 ± 2.36 (0.45)	−17.2 ± 1.29 (0.05)
PALS (%)	12 ± 6	12.89 ± 5 (0.76)	12 ± 4 (1)
Atrial Stiffness	2 ± 1	1 ± 0.66 (0.04)	1 ± 0.6 (0.04)
GWI (mmHg%)	1247 ± 591	1372 ± 423 (0.65)	1383 ± 193 (0.57)
GCW (mmHg%)	1483 ± 619	1565 ± 371 (0.76)	1629 ± 204 (0.56)
GWW (mmHg%)	162 ± 95	76 ± 35 (0.04)	64 ± 8 (0.01)
GWE (%)	88 ± 6	91 ± 1.71 (0.22)	94 ± 2 (0.02)
SAB ratio	3.3 ± 2.1	2.59 ± 1.37 (0.4)	2.17 ± 1 (0.22)
Relative apical sparing	1.3 ± 0.6	1.03 ± 0.41 (0.34)	1.02 ± 0.35 (0.30)
NT-proBNP (pg/mL)	1956 ± 3297	860 ± 1198 (0.42)	764 ± 453 (0.36)
Hs TnT (ng/L)	31 ± 48	29 ± 10 (0.91)	27 ± 9 (0.83)

## Data Availability

Data are available under request.
